# A Polyelectrolyte Colloidal Brush Based on Cellulose: Perspectives for Future Applications

**DOI:** 10.3390/polym15234526

**Published:** 2023-11-25

**Authors:** Michael A. Smirnov, Vitaly K. Vorobiov, Veronika S. Fedotova, Maria P. Sokolova, Natalya V. Bobrova, Nikolay N. Smirnov, Oleg V. Borisov

**Affiliations:** 1 Institute of Macromolecular Compounds, Russian Academy of Sciences, V.O. Bolshoi Pr. 31, 199004 St. Petersburg, Russia; vrbvrbvrb@mail.ru (V.K.V.); fedotova.veronicka2016@yandex.ru (V.S.F.); pmarip@mail.ru (M.P.S.); natalia.bobrova.60@mail.ru (N.V.B.); rambow@inbox.ru (N.N.S.); 2 Institut des Sciences Analytiques et de Physico-Chimie pour l’Environnement et les Matériaux (IPREM), UMR 5254 CNRS/UPPA, 64053 Pau, France

**Keywords:** colloidal polyelectrolyte brush, graft polymerization, cellulose nanofibers, polyacrylamide, composite hydrogel, polypyrrole, supercapacitor

## Abstract

This feature article is devoted to the evaluation of different techniques for producing colloidal polyelectrolyte brushes (CPEBs) based on cellulose nanofibers modified with grafted polyacrylates. The paper also reviews the potential applications of these CPEBs in designing electrode materials and as reinforcing additives. Additionally, we discuss our own perspectives on investigating composites with CPEBs. Herein, polyacrylic acid (PAA) was grafted onto the surface of cellulose nanofibers (CNFs) employing a “grafting from” approach. The effect of the PAA shell on the morphological structure of a composite with polypyrrole (PPy) was investigated. The performance of as-obtained CNF-PAA/PPy as organic electrode material for supercapacitors was examined. Furthermore, this research highlights the ability of CNF-PAA filler to act as an additional crosslinker forming a physical sub-network due to the hydrogen bond interaction inside chemically crosslinked polyacrylamide (PAAm) hydrogels. The enhancement of the mechanical properties of the material with a concomitant decrease in its swelling ratio compared to a pristine PAAm hydrogel was observed. The findings were compared with the recent theoretical foundation pertaining to other similar materials.

## 1. Introduction

The advantages of a wider application of cellulose-based materials as a new type of absorbents or fillers are related to their abundance, low cost, easy recycling, and biocompatibility. These properties are of special interest for application in different practical fields such as environmental protection [1,2], energy storage [3], garment design [4], surgical pads [5], wound healing [6,7,8], absorbent hygiene products [9], pharmaceuticals [10,11,12], and 3D printing [13]. Cellulose-based gels are also studied as future materials for food packaging and plant agriculture [14], antimicrobial [15], and electroactive [16] materials. The chemical structure of the material’s surface drives its interaction with an environment, which is of prime importance for attaining the desired interaction with specific molecules or turning compatibility between the material components [17]. Thus, the methods for the modification of surfaces via small organic molecules and polymer chains using the hydroxyl group chemistry are intensively elaborated, especially for the tuning of sorption properties and the stability of cellulose nanomaterials, namely cellulose nanocrystals (CNCs) and nanofibers (CNFs). Different methods, including “grafting to”, “grafting from” and “grafting through” are used to modify cellulose surfaces with polymers [18]. The resulting objects can be coined as “colloid brushes” through analogy to molecular polymer brushes (bottle brushes) that are formed by grafting the side polymer chains to the main polymer backbone. The grafting of polymer chains can be performed via different types of polymerization: free radical [19], controlled radical [20], ionic [21], and ring opening [22]. The free-radical “grafting from” polymerization of acrylic or vinyl monomers onto cellulose surface is the easiest to perform both in the laboratory and on the industrial scale because this process is tolerant to moisture; does not need organic solvents; and can be initiated using relatively cheap and abundant thermal or photo-initiators, such as ammonium or potassium peroxydisulfate [19,23,24], red-ox initiators [25], cerium ammonium nitrate [26,27], and diphenylketone [28]. The drawbacks of this method are the relatively low degree of control of the grafted polymer molecular mass distribution and the need for further purification from homopolymer (not grafted). At the same time, controlled radical polymerization such as atom transfer radical polymerization (ATRP) can achieve the fine architecture of grafts, including acrylic polymers [29,30,31,32]. For example, Hansson et al. synthesized a copolymer of methyl methacrylate and glycidyl methacrylate, which was grafted onto cellulose in order to impart hydrophobic properties to it [33]. The modification of cellulose fibers was carried out via the interaction of surface hydroxyl groups with 2-bromoisobutyryl bromide followed by grafting with poly(methylacrylate) [34] or poly(glycidylmethacrylate) [35]. However, due to the need of the employing of organic solvents for this procedure, its large-scale production is difficult to implement. Thus, the use of conventional initiators dominates in patents oriented on the industrial-scale preparation of cellulose grafted with polyacrylates [36,37].

FTIR spectroscopy is commonly used to confirm cellulose modification with acrylates. For example, absorption bands centered near 1710 cm^−1^ attributed to –COOH groups, 1730 cm^−1^ for acrylic ethers containing –COOR, and very intensive amide I band at 1670 cm^−1^ for the amide groups of polyacrylamide can be distinguished [25,38]. These bands are known to be absent in a spectrum of pure (unoxidized) cellulose.

Grafting polymers onto the cellulose surface leads to a number of practically important effects. First of all, it can be pointed out that grafting 1 wt% of polyacrylic acid (PAA) to the surface of CNCs using an (NH_4_)_2_S_2_O_8_/NaHSO_3_ initiator system results in prolonged CNC dispersion stability due to the surface charge and steric hindrance arising from grafted polymer molecules [39]. It was proposed that the stabilization effect of grafted polymers is more permanent in comparison with the common approach based on the charging of CNC surface with sulfate ester groups because the latter can be easily removed under mild alkaline conditions [40].

The stabilization of cellulose dispersions by lowering the surface tension is used to obtain its composites with hydrophobic polymers, which makes it possible to prepare cellulose-based reinforced thermoplastic composites. It was demonstrated that the grafting of poly(butyl acrylate) leads to an improvement in the mechanical characteristics of propylene-based biocomposites [41]. The introduction of CNC modified by grafted poly(methyl methacrylate) improves the elastic modulus and tensile strength of poly(butyl acrylate-co-methyl methacrylate) [42]. A CNF modified with poly(stearylacrylate) increased Young’s modulus of polyethylene from 888 MPa up to 1094 MPa [43]. Poly(methyl methacrylate) was reinforced with CNCs modified by grafting 2-isocyanatoethyl methacrylate, which led to an increase in strength from 28.7 MPa to 58.6 MPa. Also, it should be mentioned that in other cases, cellulose nanoparticles need to have enhanced hydrophilic properties to accelerate their interactions with hydrophilic polymers. Using rheological measurements [44], it was demonstrated that the grafting of PAA to the CNC surface enhances the interaction of nanoparticles with glycoprotein mucin, which is beneficial for local drug delivery in colorectal cancer.

The grafted polyacrylate chains act as the immobilized nanolayer of polymer gel on the surface of the hard cellulose backbone. This provides new sorption properties to the cellulose nanomaterials, making their behavior similar to that of bulk polyacrylate gels but with significantly enhanced surface area. As a result, diffusion processes are significantly improved, and the obtained materials are proposed to be effective sorbents for organic molecules, which are mainly used in water purification and drug delivery. Thus, cellulose nanocrystals are considered prospective drug carriers that allow for the prolonged release of therapeutic molecules. The ability of the grafted polyacrylate chains to introduce active binding centers with “not-intrinsic cellulose” chemistry onto the nanoparticles’ surface allows for the regulation of sorption activity as well as release profile. PAA-grafted CNCs demonstrated a higher release duration of cisplatin in comparison with pristine CNCs [44]. Shahzadi with coauthors [45] proposed cellulose nanocrystals modified by grafted polyacrylic acid and doped with MgO, which was successfully used as the doxorubicin delivery system. In another work [46], ATRP-prepared polymer brushes based on the grafted copolymers of cellulose and polymethacrylic acid demonstrated good efficiency in drug (porphyrazine-type) delivery in vivo.

Due to the possibility of dramatically changing cellulose surface adsorption properties, polymer-grafted CNCs are intensively studied as materials for binding metal ions or even dispersed particles that are also used for water purification. Rani et al. [47] developed an n-butyl acrylate-grafted CNC copolymer capable of removing up to ~90 wt% of Pb (II) cations from an aqueous solution (200 mg/L) using an adsorbent dosage of 1 g/100 mL. Zhao et al. [48] obtained a crosslinked hydrogel adsorbent comprising microcrystalline cellulose with a grafted copolymer of acrylic acid and acrylamide for the removal of Cu (II), Pb (II), and Cd (II) from an aqueous solution. The maximum sorption capacities for these metal ions were found to be 157.51, 393.28, and 289.97 mg/g, respectively. It should be noted that polyacrylate-based hydrogels are also being investigated for their potential in metal ion sorption, although in some cases, they have been found to be less efficient than cellulose-grafted colloids. For instance, hydrogels based on hydrolyzed polyacrylamide and aliphatic diamines exhibited sorption capacities for Cu (II) ranging from 40 to 120 mg/g [49]. In another study [50], a MnO_2_-modified biochar/polyacrylamide hydrogel demonstrated a sorption capacity of 70.90 and 84.76 mg/g compared with Pb(II) and Cd(II) ions, respectively.

One of the directions studied for the application of modified CNCs is their usage as the next generation of flocculants. The direct approach for the preparation of cellulose-based flocculants is the modification of the cellulose backbone via oxidation [51], esterification [52], and etherification [53]. This allows for the introduction of specific charged or uncharged groups that can act as binding sites for colloidal particles. However, the reported data prove that cellulose modified in this way exhibits the best capability for flocculation of kaolin either in high dosage (150 mg/L [54]) or in combination with inorganic components such as alum [55]. At the same time, CNCs with grafted polyacrylamide [56] or poly(N,N-dimethylaminoethyl methacrylate) [57] demonstrate an optimal dosage of 10 and 5 mg/L, respectively. This can be attributed to the more flexible nature of grafted polyacrylates in comparison with CNCs, which allows for cooperative binding to the kaolin particles.

Electroactive hydrogels based on conducting polymers and/or polyelectrolytes are perspective materials for various applications [58], such as energy storage, and intensively studied during the last few years [59,60,61,62]. One of the main directions in the elaboration of electrode materials with improved performance is through an increase in the specific surface area of hydrogels as a result of a rough morphology or porous structure, and for this purpose, treatments of hydrogels with organic solvent [63] or electrospinning have been applied [64]. These materials are usually prepared via the in situ polymerization of a conductive polymer inside a crosslinked matrix of hydrogel [65]. In this regard, using a gel nanolayer connected to a hard framework, for example, a CNF backbone, can be a way of increasing the electroactive hydrogel surface [66]. At the same time, the use of cellulose-based colloidal polyelectrolyte brushes (CPEBs) can enhance the selective formation of conductive polymer inside the polyelectrolyte part [67].

**Figure 1:**
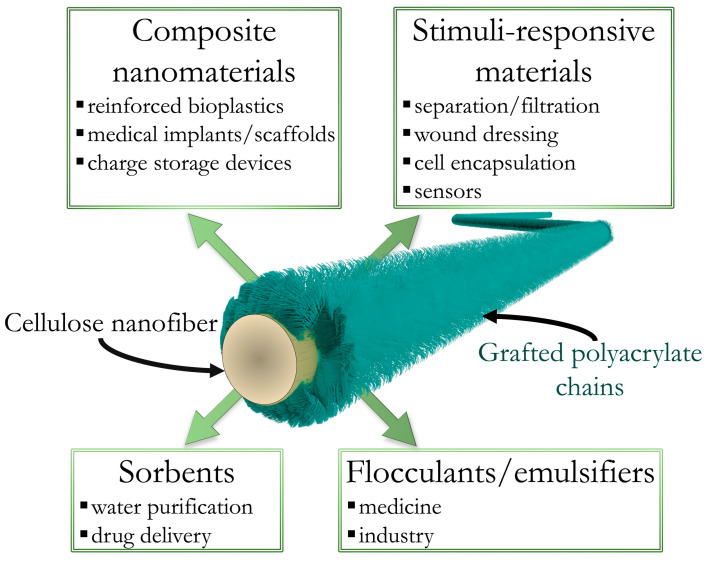
Illustration of cellulose nanofiber with a surface modified by polyacrylate and its possible fields of application.

As can be seen from the literature, there are many opportunities for the use of cellulose-based colloid brushes with adjusted surface chemistry in various applications (see Figure 1). Furthermore, we aim to propose two additional approaches that are rarely mentioned in the literature but follow the main directions of using tuned cellulose surface chemistry in the preparation of the next generation of advanced materials.

(1) The sorption properties of CNFs with grafted polyacrylic acid can be utilized for the formation of electroconductive nanofibers via the sorption of a monomer (pyrrole) that can be polymerized, giving rise to a conductive polymer (polypyrrole). Additionally, the selective polymerization inside the polyacrylate shell can be performed via the preliminary sorption of Fe^3+^ in it. This will govern the polymerization to proceed mainly inside the volume of the shell and result in the formation of fine electroconductive PAA–polypyrrole nanofibers with a hard CNF backbone.
(2) According to the literature discussed above, the enhanced interactions of CNF-PAA colloid brushes with hydrophilic polymers result in the formation of a cooperative network of hydrogen bonds that can act as crosslinks when the CNF-PAA brush is placed inside the solution containing polyacrylamide or another polymer with a polar group. The cooperative and reversible nature of such interactions, along with the high rigidity of the CNF backbone, can lead to the formation of additional sacrificial sub-networks in the hydrogel, which can facilitate an improvement in the mechanical properties of polymer hydrogels, as recently demonstrated for the application of the coordination bonds of a PAA-based polymer network with metal ions [68].

In the next sections, the experiments that aim to investigate this hypothesis are described. Finally, the theoretical consideration concerning the structure and behavior of the colloid PAA brush in the polymer network will be highlighted.

## 2. Materials and Methods

### 2.1. Materials

Choline chloride (CAS 67-48-1, purity ≥ 99%, Glentham Life Sciences Ltd., Corsham, UK) was vacuum-dried for 48 h at 60 °C prior to use. Acrylamide (CAS 79-06-1, purity ≥ 98%) and acrylic acid (CAS 79-10-7, purity ≥ 99%) were purchased from Sigma-Aldrich (Prague, Czech Republic). Acrylamide and ammonium persulfate (NH_4_)_2_S_2_O_8_ (CAS 7727-54-0, purity ≥ 98% from Vekton, Saint Petersburg, Russia) were recrystallized before use. Pyrrole (CAS 109-97-7, purity ≥ 99%) was received from Acros Organics (Geel, Belgium), and N,N′-methylenebisacrylamide (MBA) (CAS 110-26-9, purity > 99%) and N,N,N′,N′-tetramethylethane-1,2-diamine (TEMED) (CAS 110-18-9, purity > 99%) were purchased from Sigma-Aldrich (St. Louis, MO, USA). Urea (CAS 57-13-6, purity > 98%) was obtained from LenReactiv (Saint Petersburg, Russia), nitric acid HNO_3_ 65 wt% (CAS 7697-37-2) was obtained from Vekton (Saint Petersburg, Russia), and 0.1 N standard titer hydrochloric acid HCl (CAS 7647-01-0) was purchased from Acroshim (Saint Petersburg, Russia), and they were used as received. The sodium tartrate dehydrate (CAS 868-18-8, purity > 99.5%) and NaOH (CAS 1310-73-2, purity > 99%) were purchased from NevaReaktiv (Saint Petersburg, Russia). Anhydrous iron chloride (III) FeCl_3_ (CAS 7705-08-0, purity ≥ 97%, Fisher Chemical, Loughborough, UK) and cerium (IV) ammonium nitrate (CAS 16774-21-3, purity ≥ 99%, Acros Organics, Geel, Belgium) were used as received. Peptone (CAS 73049-73-7) and D-mannitol (CAS 69-65-8), obtained from LenReaktiv (Saint Petersburg, Russia), as well as yeast extract (CAS 8013-01-2), obtained from the Research Center for Pharmacotherapy (Saint Petersburg, Russia), were used to prepare the culture medium.

### 2.2. Methods

#### 2.2.1. Preparation of Polyacrylic Acid Grafted Bacterial Cellulose Nanofibers

The biosynthesis of bacterial cellulose (BC) was carried out according to the standard method published in our previous work [13]. The degree of BC polymerization according to the viscometry method was 1100 [69]. CNFs were obtained using the following procedure: A BC pellicle was ground with a laboratory blender and freeze-dried. The obtained powder was dispersed in a deep eutectic solvent based on choline chloride and urea at 115 °C for 1 h and then rinsed with water. The resulting aqueous CNF dispersion was used to obtain electrode materials after the modification of the cellulose surface with PAA and without it.

CNF-grafted PAA (CNF-PAA) was obtained via the “grafting from” approach using cerium (IV) ammonium nitrate as a reduction–oxidation (redox) initiator. For this purpose, 0.55 g of acrylic acid was added to 20 mL of 0.31 wt% aqueous CNF dispersion. Subsequently, 0.1233 g of cerium (IV) ammonium nitrate was dissolved in 2 mL of 4.7 wt% nitric acid, and the mixture was poured. The reaction was carried out with stirring without heating for 24 h. The color of the dispersion changed from orange to colorless during the reaction. To remove the homopolymer PAA, the resulting CNF-PAA was washed with distilled water through repetitive centrifugation and redispergation.

#### 2.2.2. The Preparation of Electroactive Electrode Material Based on the CNF-PAA Brush (CNF-PAA-PPy)

The CNF and CNF-PAA were modified with PPy in order to obtain electrode materials for supercapacitors. In situ synthesis was carried out on a graphite substrate with a diameter of 1.2 cm used as a working electrode. Aqueous dispersions of CNF or CNF-PAA were ultrasonicated for 30 min in the presence of ammonium persulfate (APS) or FeCl_3_ as an initiator of pyrrole polymerization. Pyrrole (2.6 mg), dissolved in 72 mg of water, was deposited on the graphite surface. After that, the CNF or CNF-PAA dispersion containing the initiator was added to the monomer solution to start pyrrole polymerization. During synthesis, polypyrrole particles were deposited and adsorbed on the CNF or CNF-PAA surface, which changed the color of the material to black. The release of the polymer was not observed during washing with water and further manipulations with prepared gel electrodes; thus, it was assumed that the obtained conductive polymer was completely incorporated into the material. The molar ratios of APS–pyrrole and FeCl_3_–pyrrole were 1:1 and 2.25:1, respectively. The cellulose content in all composites was 2% of the mass of pyrrole. Thus, the weight ratio of polypyrrole/cellulose was estimated as 50:1. The electrode samples were dried at room conditions for 12 h before measurements.

#### 2.2.3. The Preparation of CNF-PAA-Brush-Reinforced Polyacrylamide Hydrogel (CNF-PAA-PAAm)

Polyacrylamide hydrogels filled with CNF-PAA were obtained via the thermoinitiated radical polymerization of 25 wt% acrylamide (AAm) in distilled water containing MBA (1 wt% of the total mass of the monomer) as a crosslinking agent. APS and N,N,N′,N′-tetramethylethane-1,2-diamine were used as the initiating system. The molar ratio of AAm–initiator was 2500:1. The concentration of CNF-PAA in dispersion was 1 wt%. Previously, NaOH was added to the dispersion of CNF-PAA to neutralize the PAA (the amount of NaOH corresponded to 50 mol% by weight of the PAA). Polyacrylamide gel in the absence of cellulose was similarly prepared. The dispersion was filled into a polymer tube with a diameter of 4.6 mm and a height of 40 mm. The reaction was carried out for 24 h at 50 °C. The prepared reinforced hydrogels had two interpenetrating networks: a covalently crosslinked PAAm network and a physically crosslinked CNF-PAA network. In further discussion, these hydrogels will be named “reinforced hydrogels”.

#### 2.2.4. Titrimetric Characterization of PAA Content

The quantitative analysis of CNF-PAA was performed via potentiometric titration using a pH meter-ionomer Expert-001 (Econix-Expert, Moscow, Russia). Typically, a weighted portion (0.25–0.3 g) of the colloidal copolymer brush dispersion with a concentration of 3.8 wt% was taken and mixed with 40 mL of degassed distilled water and an excess of NaOH solution to fully convert the carboxylic groups of PAA to the salt form. Next, the dispersion was ultrasonicated and titrated with a standard titer of 0.1 N HCl solution (fixanal). The experiment was carried out for CNF and CNF-PAA samples.

#### 2.2.5. Fourie Transform Infrared Spectroscopy

The aqueous dispersions of CNF and CNF-PAA were dried at room conditions to obtain self-supported films. The chemical structures of the films were studied using Fourier transform infrared (FTIR) spectroscopy with an IRAffinity-1S spectrometer (Shimadzu, Kyoto, Japan) in the Attenuated Total Reflectance (ATR) mode. FTIR spectra were obtained with a resolution of 4 cm^−1^ within the range of 4000–400 cm^−1^ (the number of scans was 100).

#### 2.2.6. Scanning Electron Microscopy

The morphology of the electrode materials after drying was studied via scanning electron microscopy using a Tescan Vega III SEM microscope (Tescan, Brno-Kohoutovice, Czech Republic).

#### 2.2.7. Electrochemical Study of CNF-PAA-PPy

Electrochemical investigations were carried out using a potentiostat–galvanostat P40-X (Elins, Moscow, Russia) with a three-electrode cell equipped with a Ag/AgCl reference electrode, a platinum counter electrode, and a graphite work electrode with CNF/PPy or CNF-PAA/PPy samples on its surface. The cell was filled with 1 M Na_2_SO_4_ aqueous solution as the electrolyte. Cyclic voltammetry (CV) measurements were performed at varying scan rates in a range of 0.5–20 mV/s. The specific capacitance of the electrode material was determined from galvanostatic charge–discharge (GCD) experiment data (*C_sp_*, F/g) and CV data (*C_CV_*, F/g) using the following equation:

(1) $$C_{sp}=\frac{I\cdot ∆t}{m\cdot \left(U-∆U\right)},$$

(2) $$C_{CV}=\frac{\int i\left(U\right)dU}{2v\cdot m\cdot U},$$

where *I*, Δ*t*, *m*, *U*, and Δ*U* represent the constant discharge current, discharge time, the mass of the electrically conductive polymer (2.6 mg), potential window, and potential drop, respectively; *v* indicates the scan rate; and *∫i*(*U*)*dU* is the integral of the *CV* curve (generated current (*i*) against applied potential *U* ranging from −0.5 to 0.5 vs. Ag/AgCl).

#### 2.2.8. Measurements of Mechanical Properties of Hydrogels

The investigation of mechanical properties was performed for pristine PAAm and CNF-PAA-PAAm hydrogels. Specimens, prepared as described in Section 2.2.3, were cut into cylinders with a height ranging from 3.5 to 4 mm. A compression test was performed using an Instron 5943 universal testing machine (Instron, Norwood, MA, USA) at a traverse speed of 5 mm/min. Young’s modulus was determined by calculating the slope of the stress–strain curves in the initial linear region, specifically within 0–10% strain, as well as the entire mechanical curve. Statistical analysis was carried out based on the measurements of at least 5 samples.

#### 2.2.9. Measurements of CNF-PAA-PAAm Swelling at Various pH

The swelling ratio of hydrogels was studied at different pH values in the range of 2.22–11.94. Samples (approximately 0.025 g) were immersed in 80 mL of a solution with different pH levels. A week later, the mass of the swollen sample was determined using an AP225WD (Shimadzu, Kyoto, Japan) microbalance with an accuracy of 0.01 mg. Next, the samples were lyophilized with a Scientz-10ND lyophilic dryer (Scientz, Ningbo, China) for 24 h. The swelling ratio of each sample was calculated using the following equation:

(3) $$Q=\frac{m-m_{0}}{m_{0}},$$

where *m*_0_ is the sample mass before swelling, and *m* is the sample mass after swelling.

## 3. Results and Discussion

### 3.1. Characterization of Structure of CNF-PAA

**Figure 2:**
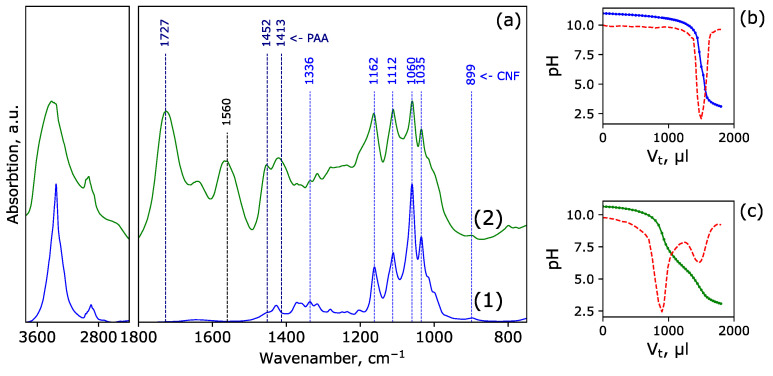
FTIR spectra of native CNF (1) and CNF-PAA (2) (**a**); potentiometric titration curve of CNF (**b**) and CNF-PAA (**c**) (solid line) and its first derivative (dashed line).

The IR spectra for pristine CNF and CNF-PAA are presented in Figure 2a. An intense band associated with the C=O group vibration at 1727 cm^−1^, as well as a noticeable band near 1560 cm^−1^, corresponding to –COO^−^, are observed, which are characteristic of PAA. At the same time, the bands associated with the presence of cellulose (1336, 1162, 1112, 1060, and 1035 cm^−1^) are retained in the CNF-PAA sample. This confirms the successful grafting of the PAA onto the surface of the CNF.

The potentiometric titration curve for the CNF and CNF-PAA samples and their first derivative are shown in Figure 2b,c, respectively. In contrast to the pristine CNF (Figure 2b), the results for the CNF-PAA sample (Figure 2c) show two well-distinguished equivalence points on the curve: the neutralization of free hydroxyl ions and a weak transition corresponding to the complete protonation of carboxyl groups. The calculated content of the grafted PAA in CNF-PAA was 41 ± 3 wt%.

### 3.2. The Morphology and Electrochemical Performance of the Electroactive Electrode Material CNF-PAA/PPy

**Figure 3:**
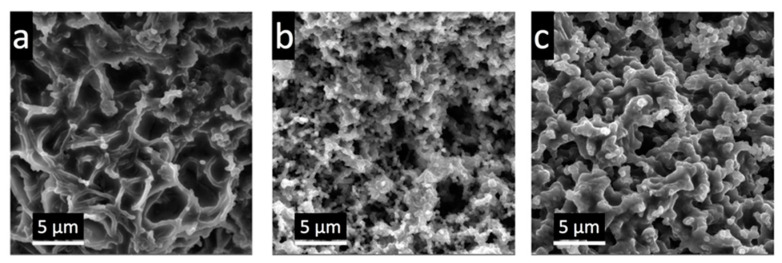
SEM images of the surface of CNF/PPy (**a**) and CNF-PAA/PPy (**b**) dried electrodes synthesized using FeCl_3_ as an initiator; SEM images of CNF-PAA/PPy electrode surface synthesized using (NH_4_)_2_S_2_O_8_ (**c**).

Figure 3 shows the SEM images of the surface of electrodes synthesized using FeCl_3_ as an initiator on the CNF and CNF-PAA (Figure 3a,b, respectively) as well as the electrode based on the CNF-PAA and prepared with (NH_4_)_2_S_2_O_8_ as the initiator (Figure 3c). The structural elements of PPy obtained using the FeCl_3_ initiator are smaller and more strongly connected to cellulose nanofibers in the case of CNF-PAA (Figure 3b) than the native CNF (Figure 3a). This demonstrates the predominant formation of PPy on the surface of the CNF modified via the grafting of PAA. This may be caused by the adsorption of FeCl_3_ (and subsequently the sorption of the pyrrole monomer) on the CNF-PAA surface, leading to a more uniform distribution of the initiation centers of polymerization over the CNF surface and the reduced probability of the formation of PPy, which is not connected to the CNF. It is worth mentioning that, under the same synthesis conditions, the electrode materials with CNF-PAA (Figure 3c) have a notably lower degree of agglomeration than those with the native CNF (Figure 3a). The effect of the sorption of the initiator in the gel nanolayer on the CNF can also be observed by changing the initiator from FeCl_3_ to the (NH_4_)_2_S_2_O_8_. In this case, the initiator ions are negatively charged, which is not favorable for their sorption inside the PAA layer. As seen in Figure 3c, the size of PPy structural elements and the size of pores increase in comparison with the polymer synthesized on the CNF-PAA using FeCl_3_. This can lead to a smaller surface accessible for electrochemical fast-charging storage reactions, which will be discussed further. This result confirms the hypothesis indicated in the Introduction section, namely the positive role of the selective sorption of reaction components on the CNF-PAA colloid brush for the formation of beneficial morphology.

**Figure 4:**
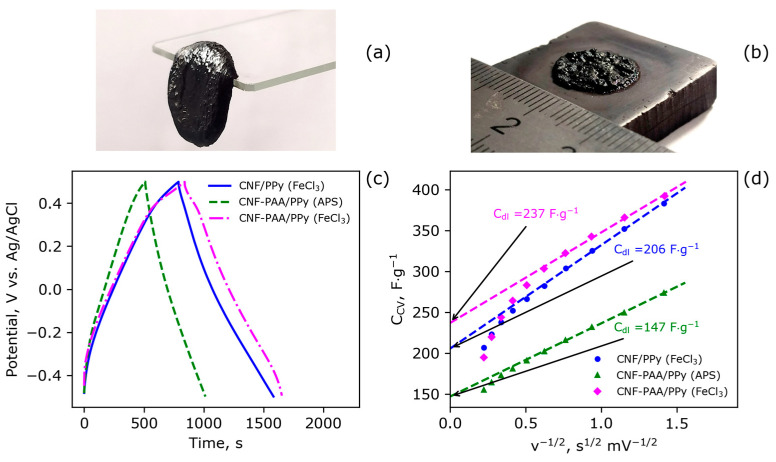
A photograph of CNF-PAA/PPy composite electrode material (**a**) and a photograph of this electrode synthesized in situ on a graphite current collector (**b**); GCD curves at the current density of 0.5 A/g from −0.5 to 0.5 V vs. Ag/AgCl using 1 M Na_2_SO_4_ as electrolyte at pH = 4–4.5 (**c**); the dependence of the specific capacitance on the square root of the reciprocal scan rate (**d**). The initiator used in the synthesis is given in brackets to the signatures.

The PPy-based hydrogel materials, when infused with the CNF and CNF-PAA, are able to retain their structural integrity due to the formation of a robust nanofiber framework (see Figure 4a). Moreover, through in situ synthesis, these materials demonstrate good adhesion to the current collector, as shown in Figure 4b. The effect of CNF modification with PAA and the effect of the type of initiator on the electrochemical characteristics of the resulting electrode materials were studied. Typical GCD curves are shown in Figure 4c. The CV curves at different scan rates are shown in Figure S1 in the Supplementary Materials. The specific capacitance values for the electrodes were calculated from the GCD data and are listed in Table 1. The electrical double-layer capacitance (*C_dl_*) for the electrodes was calculated using the CV data measured at various scan rates according to a method described by Trasatti et al. [70]. The dependences of the specific capacitance on the reciprocal square root of scanning speed are illustrated in Figure 4d. The linear regions of the dependences were extrapolated to x = 0. This approach allows for the estimation of the capacitance value at a scan rate tending toward infinity (Figure 4d), which corresponds to the very fast process that can occur only on a surface with an electrochemical double layer. The electrical double-layer capacitance is related to the specific surface area of the electrode material. The capacitances found from the GCD and Trasatti measurements are given in Table 1. It is seen that the electrode based on the CNF-PAA and prepared with FeCl_3_ has the highest capacitance. The difference in the *C_dl_* value between the electrodes based on the CNF and CNF-PAA is larger than the difference in their specific capacitance. This demonstrates the effect of a more porous electrode morphology in the case of CNF-PAA-based electrodes. The morphology and porous structure of the electrode have a more significant impact on the electrochemical performance at higher rates.

The faster decrease in capacitance with an increase in the scanning rate for the sample based on the pristine CNF in comparison with that of the modified one is also seen from the higher slope of the dependence of *C* on *v*^−1/2^. The magnitude of the difference is not very impressive, which can be attributed to the possibility that Fe^3+^ ions also sorb on the unmodified CNF surface [71,72], while the efficiency of this sorption is lower than that in the CNF grafted with PAA. However, this difference proves the hypothesis about the positive effect of CEPBs on the electrochemical performance of electrode materials. Moreover, it is clearly seen that changing the initiator to (NH_4_)_2_S_2_O_8_ leads to a significant decrease in electrochemical performance (see Table 1). These observations provide additional evidence for the importance of the initiator and monomer sorption on the template, namely the CNF in our case.

The gravimetric specific capacitance of the as-obtained CNF-PAA/PPy electrodes was found to be higher than that of other porous hydrogel electrodes with PPy reported in the literature, such as crosslinked PAAm filled with carboxymethylcellulose (CMC)/PPy (~140 F/g at 0.5 A/g) [73], a carboxylated CNF/PPy film electrode (~180 F/g at 0.5 A/g) [74], a CMC-PANI/carbon nanotube film electrode (348.8 F/g at 0.5 A/g) [75], and a porous, pure polypyrrole hydrogel prepared via the ice-templating method (265 F/g at 1.35 A/g) [76]. This comparison demonstrates the possible practical interest in electrodes based on colloid brushes. However, further investigation needs to be carried out for the optimization of preparation conditions in order to reach maximal electrochemical performance.

### 3.3. The Mechanical Properties of Reinforced Polyacrylamide Hydrogel PAAm/CNF-PAA

**Figure 5:**
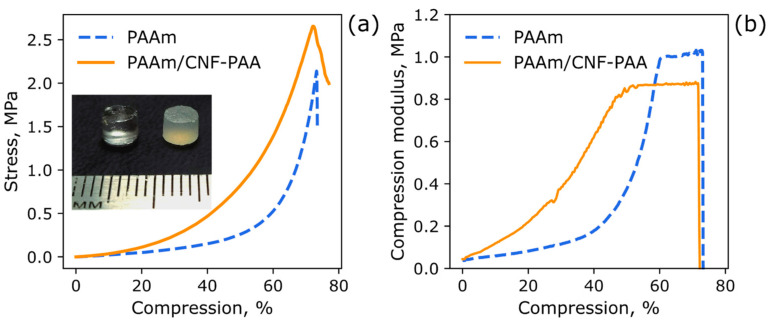
Typical stress–strain curves of crosslinked and composite hydrogels with the swelling ratio of 3 and 2.8 g/g in compression at a traverse speed of 5 mm/min (**a**) and the dependence of modulus on deformation (**b**). An inserted photograph of the pristine PAAm (**left**) and PAAm/CNF-PAA (**right**) hydrogels.

For the case of hydrogels based on the CNF-PAA brush and crosslinked PAAm, the mechanical properties and swelling at different pH levels were investigated. An inserted photograph showcasing the comparison between the pristine PAAm hydrogel (transparent) and the PAAm/CNF-PAA hydrogel (turbid) specimens is provided in Figure 5a for visual reference. The results of the mechanical measurements are given in Figure 5, while the values of the mechanical properties are given in Table 2. It is seen in Figure 5a that the stress–strain curve for the PAAm/CNF-PAA sample is above that curve for the pure PAAm, which indicates higher strength and elastic modulus for the composite hydrogel. The addition of 1 wt% of CPEBs resulted in an increase in Young’s modulus and strength from 150 to 400 kPa and from 1.9 to 2.4 MPa, respectively.

It is known that the elastic modulus for PAAm hydrogels has a nonlinear dependence on compression, while defomation is reversible at compression rates below the destruction limit for chemically crosslinked gels. Therefore, for the composite gels, several values for the elastic modulus are given corresponding to the different regions of compression [77]. Another method that can be applicable to monocomponent gels is linearization with the presentation of the compression axis as −(λ − λ^−2^), where λ is the deformation ratio (deformed length/initial length) [78]. Here, we analyzed the whole curve of the dependence of differential stress relative to compression in order to evaluate the changes in stiffness under the load for the case of the composite hydrogel. It is seen in Figure 5b that, for the pristine PAAm hydrogel, the stiffness rises exponentially and has a plateau at high compression. In the case of the PAAm/CNF-PAA sample, the increase in stiffness starts from the beginning and has less curvature and a prolonged plateau at high compression rates > 40%. This result demonstrates that the modification of the CNF even to a small extent results in the formation of an additional network inside the hydrogel with higher stiffness, which can be attributed to the interactions between PAAm and CNF-PAA or to the osmotic effect of the counterions in the grafted PAA that is partly neutralized before the preparation of hydrogels. This result supports the hypothesis presented in the Introduction section regarding the enhancement in the mechanical properties of hydrogels via the formation of an additional network based on the interactions between the CNF-PAA, as well as the CNF-PAA with PAAm, and the polymers.

In order to deeply understand the behavior of CPEBs inside the hydrogel, modern theoretical considerations are needed in the analysis. CPEBs are formed via ionically charged polymer chains covalently attached with a terminal segment to the surface of colloidal particles and immersed into a solvent. At sufficiently high surface coverage by tethered polymers, intermolecular interactions dominate over intramolecular ones. In an aqueous (or another polar) environment, the repulsive ionic interactions between the layers of ionically charged chains operating at sufficiently low ionic strength assure the aggregative stability of the polyelectrolyte-modified colloids and significantly affect the rheological properties of the dispersions.

CPEBs are classified according to the shape of the particles (e.g., spherical, cylindrical, etc.) to which the polyelectrolyte chains are grafted. Furthermore, one distinguishes CPEBs formed by either strong (pH-insensitive) polyelectrolytes, like polystyrenesulfonate, or weak (pH-sensitive) polyelectrolytes, like PAA. The structural, electrochemical, and nanomechanical properties of CPEBs and, eventually, the aggregative stability and rheological properties of their dispersions are controlled through the interplay of environmental conditions (pH, the ionic strength of the solution, etc.), as well as the architectural parameters of CPEBs (the size and shape of the particles, the polymerization degree and grafting density of the polyelectrolyte (PE) chains, etc.).

The most essential feature of CPEBs is their ability to retain a major fraction of the counterions that are inevitably present even in salt-free solutions due to the total electroneutrality condition in the interior volume of the brush [79,80]. The extent of counterion condensation or the fraction of the minority of counterions released into the solution and thus becoming osmotically active depends on the structural parameters of the brush and first and foremost on its geometry.

The theory of cylindrical CPEBs was developed by Ross R.S. and Pincus P. [81] as well as Borisov O.V. and Zhulina E.B. [82,83]. This theory considered the case that the brush thickness *D* controlled with the extension of side chains is much larger than the radius *R* of the cylindrical core, *D >> R*. It was demonstrated that, at a low salt concentration, the thickness of the brush is virtually independent of the radius *R* of the cylindrical core to which the polyelectrolyte chains are grafted and is determined (with the accuracy of the numerical prefactor on the order of unity) as *D ≈ Nα*^1/2^, where *N* is the polymerization degree of the brush-forming chains, and *α* is the fraction of ionized monomer units. Notably, this power law dependence is characteristic for any CPEB at low salt concentrations irrespective of their morphologies [81,82].

For a long and stiff cylindrical CPEB with the length *L* significantly exceeding the cross-sectional dimensions *D*, the fraction of the counterions released can be estimated using the Manning rule [84]: There is one released (monovalent) counterion per segment of the CPEB with length *l_B_*, where *l_B_* = *e*^2^/*εk_B_T* is the Bjerrum length, *e* is the elementary charge, *ε* is the dielectric permittivity of the medium, *k_B_* is the Boltzmann constant, and *T* is the temperature. If *s* is the area of the cylindrical particle per chain, the axial distance between neighboring chains is *h* = *s*/(2*πR*), and the “bare” charge (measured in the elementary charge units) per unit length of the brush is *q* = 2*π RαN*/*s*. The fraction of the released (osmotically active) counterions is thus *q_eff_*/*q* = *s*/(2*πl_B_RαN*) << 1.

Let *L* be the contour length of the cylindrical CPEB and *l_p_* be its persistence length, i.e., the brush keeps a fairly straight configuration on the length scale smaller than *l_p_*. In the general case, both the intrinsic rigidity of the cylindrical core and the induced electrostatic persistence length arising due to the enhanced Coulomb repulsions upon the bending of the CPEB contribute to *l_p_*. In the case considered here, i.e., PAA brushes grafted onto the CNF core, the former contributes more than the latter, so that *l_p_* is on the order of 10^2^ nm. If *c_cnf_* is the concentration of the CPEBs, which corresponds to the concentration of the grafted PAA chains, which is *c_PAA_* = *c_cnf_L*/*h*, then the concentration of osmotically active counterions can be estimated as *c_ions_* ≈ *c_cnf_L*/*l_B_*. Hence, the osmotic pressure generated by counterions in the solution of CPEBs and normalized by *k_B_T* can be estimated as *P_ions_*/*k_B_T* ≈ *c_cnf_L*/*l_B_*.

In order to estimate the relative contribution of the counterions to the cumulative osmotic pressure (and bulk osmotic modulus) of the PAAm crosslinked network with embedded CPEBs, the latter value has to be compared to *ξ*^−3^, where *ξ* is the average distance between the crosslinking points in the swollen PAAm gel. As shown below, in our system, the counterions’ contribution is negligible, and thus the counterions released by CPEBs do not contribute to the equilibrium swelling or the osmotic modulus of the interpenetrating network, which, as a consequence, are independent of the ionization state of the CPEB forming chains.

On the other hand, the incorporation of the CPEB into the PAAm network may lead to the appearance of additional crosslinking points due to, e.g., the formation of hydrogen bonds, which may lead to a decrease in the equilibrium swelling ratio compared to that of a pure PAAm gel. This prediction is in line with experimental observations, as demonstrated below.

**Figure 6:**
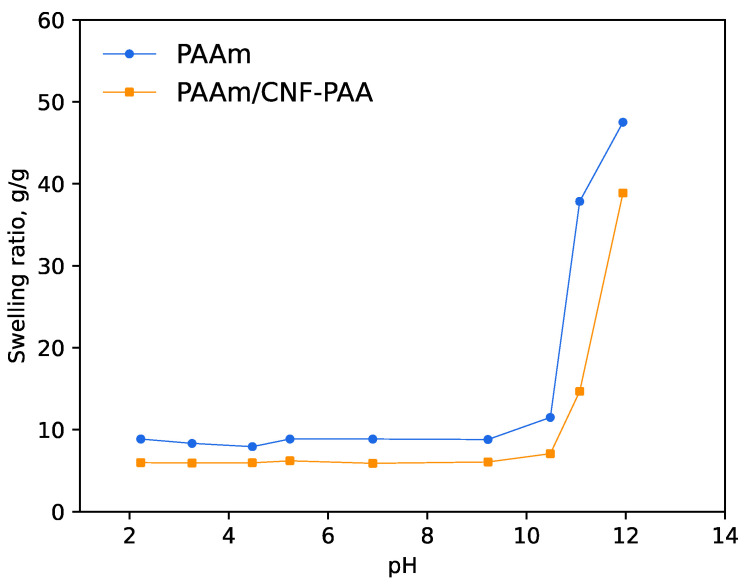
Swelling of PAAm hydrogel and PAAm hydrogel prepared in the presence of 50% charged CNF-PAA brush at different pH levels at room temperature.

To further evaluate the discussed findings, the swelling of PAAm and PAAm/CNF-PAA hydrogels was studied at different pH levels (see Figure 6). It is seen that the addition of the brush only results in a decrease in the swelling degree, while the overall course of the curve is the same for both samples. Thus, it seems that the introduction of CNF-PAAm leads to the appearance of additional crosslinks, while the ionogenic and pH-sensitive properties of PAA do not have an impact on the properties of the material. This observation confirms the theoretical considerations discussed above.

## 4. Conclusions

Cellulose nanofibers (CNFs) with grafted polyacrylic acid (CNF-PAA) have been successfully obtained using the “grafting from” method. The potential applications of colloidal polyelectrolyte brushes (CPEBs) in the field of electronics, specifically as electrode materials for supercapacitors, as well as reinforcing additives, have been explored. Remarkably, the CNF-PAA-based electrode material, when combined with polypyrrole (PPy), exhibited the highest specific capacitance compared with that prepared with the native CNF primarily due to having the largest surface area (in terms of the developed porous structure), leading to a higher double-layer capacitance. This supports the hypothesis that the sorption of iron ions as initiators on the grafted PAA shell promotes the prevailing formation of PPy on the CPEBs’ surface and its even distribution. 

The incorporation of just 1 wt% of CPEBs led to a significant increase of about 60% in the elastic modulus and 25% in the compressive strength of the polyacrylamide (PAAm) hydrogel. This can be associated with the additional rigid CNF-PAA network, which effectively interacts with the PAAm matrix through cooperative H-bonds and the formation of interpolymer complexes. Nevertheless, based on the swelling behavior of the pristine and composite hydrogels, their mechanical characteristics are largely conditioned by hydrogen bonding. Overall, these findings provide valuable insights and recommendations for the further advancement of CPEB-based composites with desired properties.

## Figures and Tables

**Table 1:** Electrochemical characteristics and the swelling ratio (Q) of supercapacitors obtained via the in situ polymerization of electrodes.

Sample	Initiator	*C_sp_* ^1^, F/g	*C_dl_*, F/g	*Q*, g/g
CNF/PPy	FeCl_3_	401	206	6.7
CNF-PAA/PPy	FeCl_3_	414	237	6.2
CNF-PAA/PPy	APS	254	147	6.5

^1^ Specific capacitance (*C_sp_*) calculated from the galvanostatic discharge curve at the current density of 0.5 A/g.

**Table 2:** Mechanical characteristics for samples.

Sample	Young’s Modulus, kPa (ε ^1^ = 0–10%)	Compression Strength, MPa	Compression at Break, %
PAAm	150 ± 40	1.9 ± 0.2	76 ± 2
PAAm/CNF-PAA	400 ± 60	2.4 ± 0.2	73 ± 2

^1^ ε—deformation (%).

## Data Availability

Data are contained within the article and Supplementary Materials.

## Supplementary Materials

The following supporting information can be downloaded at: https://www.mdpi.com/article/10.3390/polym15234526/s1, Figure S1: Cyclic voltammetry curves for CNF/PPy (a) and CNF-PAA/PPy (b) electrodes synthesized using FeCl_3_ as an initiator and CNF-PAA/PPy electrode synthesized using (NH_4_)_2_S_2_O_8_ at different scan rates (c).

## References

[1] Yang J., Han X., Yang W., Hu J., Zhang C., Liu K., Jiang S. (2023). Nanocellulose-based composite aerogels toward the environmental protection: Preparation, modification and applications. Environ. Res..

[2] Ning L., You C., Jia Y., Chen J., Zhang Y., Li X., Rojas O.J., Wang F. (2023). Cellulose nanocrystals for crop protection: Leaf adhesion and controlled delivery of bioactive molecules. Green Chem..

[3] Dong J., Li P., Zeng J., Wang B., Gao W., Xu J., Chen K. (2023). Lignin/polypyrrole interpenetrating networks decorated Lignin-containing cellulose nanofibril composite membrane for High-performance supercapacitors. Chem. Eng. J..

[4] Su S., Hu S., Liu Q. (2022). Application of Polypyrrole Cellulose Nanocrystalline Composite Conductive Material in Garment Design. Adv. Mater. Sci. Eng..

[5] Lustig M., Gefen A. (2022). The biomechanical efficacy of a dressing with a soft cellulose fluff core in protecting prone surgical patients from chest injuries on the operating table. Int. Wound J..

[6] Zheng L., Li S., Luo J., Wang X. (2020). Latest Advances on Bacterial Cellulose-Based Antibacterial Materials as Wound Dressings. Front. Bioeng. Biotechnol..

[7] Hakkarainen T., Koivuniemi R., Kosonen M., Escobedo-Lucea C., Sanz-Garcia A., Vuola J., Valtonen J., Tammela P., Mäkitie A., Luukko K. (2016). Nanofibrillar cellulose wound dressing in skin graft donor site treatment. J. Control. Release.

[8] Blažic R., Marušić K., Vidović E. (2023). Swelling and Viscoelastic Properties of Cellulose-Based Hydrogels Prepared by Free Radical Polymerization of Dimethylaminoethyl Methacrylate in Cellulose Solution. Gels.

[9] Enawgaw H., Tesfaye T., Yilma K.T., Limeneh D.Y. (2021). Synthesis of a Cellulose-Co-AMPS Hydrogel for Personal Hygiene Applications Using Cellulose Extracted from Corncobs. Gels.

[10] Khojastehfar A., Mahjoub S. (2021). Application of Nanocellulose Derivatives as Drug Carriers; A Novel Approach in Drug Delivery. Anticancer. Agents Med. Chem..

[11] Ciolacu D.E., Nicu R., Ciolacu F. (2020). Cellulose-Based Hydrogels as Sustained Drug-Delivery Systems. Materials.

[12] Xing R., Ning L., Li L., He L., Lin H., You C., Wang F. (2023). Efficient in vitro delivery of paclitaxel by a nanocellulose-coated dendritic mesoporous organosilica nanoparticle for enhanced chemodynamic cancer therapy. J. Drug Deliv. Sci. Technol..

[13] Smirnov M.A., Fedotova V.S., Sokolova M.P., Nikolaeva A.L., Elokhovsky V.Y., Karttunen M. (2021). Polymerizable choline-and imidazolium-based ionic liquids reinforced with bacterial cellulose for 3d-printing. Polymers.

[14] Cao X., Li F., Zheng T., Li G., Wang W., Li Y., Chen S., Li X., Lu Y. (2022). Cellulose-based functional hydrogels derived from bamboo for product design. Front. Plant Sci..

[15] Turky G., Moussa M.A., Hasanin M., El-Sayed N.S., Kamel S. (2021). Carboxymethyl Cellulose-Based Hydrogel: Dielectric Study, Antimicrobial Activity and Biocompatibility. Arab. J. Sci. Eng..

[16] El-Sayed N.S., Moussa M.A., Kamel S., Turky G. (2019). Development of electrical conducting nanocomposite based on carboxymethyl cellulose hydrogel/silver nanoparticles@polypyrrole. Synth. Met..

[17] Long W., Ouyang H., Hu X., Liu M., Zhang X., Feng Y., Wei Y. (2021). State-of-art review on preparation, surface functionalization and biomedical applications of cellulose nanocrystals-based materials. Int. J. Biol. Macromol..

[18] Liyanage S., Acharya S., Parajuli P., Shamshina J.L., Abidi N. (2021). Production and Surface Modification of Cellulose Bioproducts. Polymers.

[19] Kumar M., Gehlot P.S., Parihar D., Surolia P.K., Prasad G. (2021). Promising grafting strategies on cellulosic backbone through radical polymerization processes—A review. Eur. Polym. J..

[20] Garcia-Valdez O., Champagne P., Cunningham M.F. (2018). Graft modification of natural polysaccharides via reversible deactivation radical polymerization. Prog. Polym. Sci..

[21] Wan W., Ouyang H., Long W., Yan W., He M., Huang H., Yang S., Zhang X., Feng Y., Wei Y. (2019). Direct Surface Functionalization of Cellulose Nanocrystals with Hyperbranched Polymers through the Anionic Polymerization for pH-Responsive Intracellular Drug Delivery. ACS Sustain. Chem. Eng..

[22] Olsén P., Herrera N., Berglund L.A. (2020). Polymer Grafting Inside Wood Cellulose Fibers by Improved Hydroxyl Accessibility from Fiber Swelling. Biomacromolecules.

[23] Kumar R., Sharma R.K., Singh A.P. (2019). Grafting of cellulose with N-isopropylacrylamide and glycidyl methacrylate for efficient removal of Ni(II), Cu(II) and Pd(II) ions from aqueous solution. Sep. Purif. Technol..

[24] Khalilzadeh M.A., Hosseini S., Rad A.S., Venditti R.A. (2020). Synthesis of Grafted Nanofibrillated Cellulose-Based Hydrogel and Study of Its Thermodynamic, Kinetic, and Electronic Properties. J. Agric. Food Chem..

[25] Thakur V.K., Thakur M.K., Gupta R.K. (2013). Rapid synthesis of graft copolymers from natural cellulose fibers. Carbohydr. Polym..

[26] Liu X., Wen Y., Qu J., Geng X., Chen B., Wei B., Wu B., Yang S., Zhang H., Ni Y. (2019). Improving salt tolerance and thermal stability of cellulose nanofibrils by grafting modification. Carbohydr. Polym..

[27] Mzinyane N.N., Chiririwa H., Ofomaja A.E., Naidoo E.B. (2019). Effect of ammonium ceric nitrate as initiator in grafting of acrylic acid onto pine cone powder. Cellul. Chem. Technol..

[28] Kumar R., Sharma R.K., Singh A.P. (2018). Grafted cellulose: A bio-based polymer for durable applications. Polym. Bull..

[29] Grishkewich N., Akhlaghi S.P., Zhaoling Y., Berry R., Tam K.C. (2016). Cellulose nanocrystal-poly(oligo(ethylene glycol) methacrylate) brushes with tunable LCSTs. Carbohydr. Polym..

[30] Hatton F.L., Kedzior S.A., Cranston E.D., Carlmark A. (2017). Grafting-from cellulose nanocrystals via photoinduced Cu-mediated reversible-deactivation radical polymerization. Carbohydr. Polym..

[31] Yuan W., Wang C., Lei S., Chen J., Lei S., Li Z. (2018). Ultraviolet light-, temperature- and pH-responsive fluorescent sensors based on cellulose nanocrystals. Polym. Chem..

[32] Boujemaoui A., Cobo Sanchez C., Engström J., Bruce C., Fogelström L., Carlmark A., Malmström E. (2017). Polycaprolactone Nanocomposites Reinforced with Cellulose Nanocrystals Surface-Modified via Covalent Grafting or Physisorption: A Comparative Study. ACS Appl. Mater. Interfaces.

[33] Hansson S., Östmark E., Carlmark A., Malmström E. (2009). ARGET ATRP for Versatile Grafting of Cellulose Using Various Monomers. ACS Appl. Mater. Interfaces.

[34] Carlmark A., Malmström E.E. (2003). ATRP Grafting from Cellulose Fibers to Create Block-Copolymer Grafts. Biomacromolecules.

[35] Nyström D., Lindqvist J., Östmark E., Hult A., Malmström E. (2006). Superhydrophobic bio-fibre surfaces via tailored grafting architecture. Chem. Commun..

[36] Castro D.J., Karnati R., Wilson S.M., Cheng W., Liu M., Zhang Z. (2013). Use of Nanocrystaline Cellulose and Polymer Grafted Nanocrystaline Cellulose for Increasing Retention, Wet Strength, and Dry Strength in Papermaking. Process. Patent.

[37] Zhang W., Zhao J.Q., Lu C.H. (2015). Cellulose Nano-Fibrous Hyperbranched Method of. Modifying. Patent.

[38] Moghaddam P.N., Avval M.E., Fareghi A.R. (2014). Modification of cellulose by graft polymerization for use in drug delivery systems. Colloid Polym. Sci..

[39] Cheng D., Wen Y., An X., Zhu X., Cheng X., Zheng L., E Nasrallah J. (1996). Improving the colloidal stability of Cellulose nano-crystals by surface chemical grafting with polyacrylic acid. J. Bioresour. Bioprod..

[40] Habibi Y., Chanzy H., Vignon M.R. (2006). TEMPO-mediated surface oxidation of cellulose whiskers. Cellulose.

[41] Li S., Xiao M., Zheng A., Xiao H. (2011). Cellulose Microfibrils Grafted with PBA via Surface-Initiated Atom Transfer Radical Polymerization for Biocomposite Reinforcement. Biomacromolecules.

[42] Zhang J., Li M.-C., Zhang X., Ren S., Dong L., Lee S., Cheng H.N., Lei T., Wu Q. (2019). Surface modified cellulose nanocrystals for tailoring interfacial miscibility and microphase separation of polymer nanocomposites. Cellulose.

[43] Dalloul F., Mietner J.B., Navarro J.R.G. (2022). Production and 3D Printing of a Nanocellulose-Based Composite Filament Composed of Polymer-Modified Cellulose Nanofibrils and High-Density Polyethylene (HDPE) for the Fabrication of 3D Complex Shapes. Fibers.

[44] Vakili M.R., Mohammed-Saeid W., Aljasser A., Hopwood-Raja J., Ahvazi B., Hrynets Y., Betti M., Lavasanifar A. (2021). Development of mucoadhesive hydrogels based on polyacrylic acid grafted cellulose nanocrystals for local cisplatin delivery. Carbohydr. Polym..

[45] Shahzadi I., Islam M., Saeed H., Haider A., Shahzadi A., Haider J., Ahmed N., Ul-Hamid A., Nabgan W., Ikram M. (2022). Formation of biocompatible MgO/cellulose grafted hydrogel for efficient bactericidal and controlled release of doxorubicin. Int. J. Biol. Macromol..

[46] Krasnopeeva E.L., Melenevskaya E.Y., Klapshina L.G., Shilyagina N.Y., Balalaeva I.V., Smirnov N.N., Smirnov M.A., Yakimansky A.V. (2021). Poly(methacrylic Acid)-Cellulose Brushes as Anticancer Porphyrazine Carrier. Nanomaterials.

[47] Rani K., Gomathi T., Vijayalakshmi K., Saranya M., Sudha P.N. (2019). Banana fiber Cellulose Nano Crystals grafted with butyl acrylate for heavy metal lead (II) removal. Int. J. Biol. Macromol..

[48] Zhao B., Jiang H., Lin Z., Xu S., Xie J., Zhang A. (2019). Preparation of acrylamide/acrylic acid cellulose hydrogels for the adsorption of heavy metal ions. Carbohydr. Polym..

[49] Jumadilov T., Malimbayeva Z., Yskak L., Suberlyak O., Kondaurov R., Imangazy A., Agibayeva L., Akimov A., Khimersen K., Zhuzbayeva A. (2022). Comparative Characteristics of Polymethacrylic Acid Hydrogel Sorption Activity in Relation to Lanthanum Ions in Different Intergel Systems. Chem. Chem. Technol..

[50] Wu Z., Chen X., Yuan B., Fu M.-L. (2020). A facile foaming-polymerization strategy to prepare 3D MnO2 modified biochar-based porous hydrogels for efficient removal of Cd(II) and Pb(II). Chemosphere.

[51] Grenda K., Arnold J., Gamelas J.A.F., Cayre O.J., Rasteiro M.G. (2020). Flocculation of silica nanoparticles by natural, wood-based polyelectrolytes. Sep. Purif. Technol..

[52] Aloulou F., Boufi S., Labidi J. (2006). Modified cellulose fibres for adsorption of organic compound in aqueous solution. Sep. Purif. Technol..

[53] Kono H. (2017). Cationic flocculants derived from native cellulose: Preparation, biodegradability, and removal of dyes in aqueous solution. Resour. Technol..

[54] Quinlan P.J., Tanvir A., Tam K.C. (2015). Application of the central composite design to study the flocculation of an anionic azo dye using quaternized cellulose nanofibrils. Carbohydr. Polym..

[55] Nourani M., Baghdadi M., Javan M., Bidhendi G.N. (2016). Production of a biodegradable flocculant from cotton and evaluation of its performance in coagulation-flocculation of kaolin clay suspension: Optimization through response surface methodology (RSM). J. Environ. Chem. Eng..

[56] Zhang H., Guo H., Wang B., Xiong L., Shi S., Chen X. (2016). Homogeneous synthesis and characterization of polyacrylamide-grafted cationic cellulose flocculants. J. Appl. Polym. Sci..

[57] Parviainen H., Hiltunen M., Maunu S.L. (2014). Preparation and flocculation behavior of cellulose-g-PMOTAC copolymer. J. Appl. Polym. Sci..

[58] Mir A., Kumar A., Riaz U. (2022). A short review on the synthesis and advance applications of polyaniline hydrogels. RSC Adv..

[59] Du J., Zhu W., Yang Q., She X., Wu H., Tsou C., Manuel D.G., Huang H. (2022). Strong conductive hybrid hydrogel electrode based on inorganic hybrid crosslinking. Colloid Polym. Sci..

[60] He X., Zhuang T., Ruan S., Xia X., Xia Y., Zhang J., Huang H., Gan Y., Zhang W. (2023). An innovative poly(ionic liquid) hydrogel-based anti-freezing electrolyte with high conductivity for supercapacitor. Chem. Eng. J..

[61] Zou Y., Zhang Z., Zhong W., Yang W. (2018). Hydrothermal direct synthesis of polyaniline, graphene/polyaniline and N-doped graphene/polyaniline hydrogels for high performance flexible supercapacitors. J. Mater. Chem. A.

[62] Ye J., Shi D., Yang Z., Chen M. (2018). Interpenetrating Network Hydrogels based on Nanostructured Conductive Polymers for Flexible Supercapacitor. Polym. Sci. Ser. A.

[63] Smirnov M.A., Sokolova M.P., Geydt P., Smirnov N.N., Bobrova N.V., Toikka A.M., Lahderanta E. (2017). Dual doped electroactive hydrogelic fibrous mat with high areal capacitance. Mater. Lett..

[64] Smirnov M.A., Tarasova E.V., Vorobiov V.K., Kasatkin I.A., Mikli V., Sokolova M.P., Bobrova N.V., Vassiljeva V., Krumme A., Yakimanskiy A.V. (2019). Electroconductive fibrous mat prepared by electrospinning of polyacrylamide-g-polyaniline copolymers as electrode material for supercapacitors. J. Mater. Sci..

[65] Smirnov M.A., Bobrova N.V., Dmitriev I.Y., Bukolšek V., Elyashevich G.K. (2011). Electroactive hydrogels based on poly(acrylic acid) and polypyrrole. Polym. Sci. Ser. A.

[66] Tu C.-W., Tsai F.-C., Chen J.-K., Wang H.-P., Lee R.-H., Zhang J., Chen T., Wang C.-C., Huang C.-F. (2020). Preparations of Tough and Conductive PAMPS/PAA Double Network Hydrogels Containing Cellulose Nanofibers and Polypyrroles. Polymers.

[67] Zheng W., Fan L., Meng Z., Zhou J., Ye D., Xu W., Xu J. (2024). Flexible quasi-solid-state supercapacitors for anti-freezing power sources based on polypyrrole@cation-grafted bacterial cellulose. Carbohydr. Polym..

[68] Liu T., Chen W., Li K., Long S., Li X., Huang Y. (2023). Toughening Weak Polyampholyte Hydrogels with Weak Chain Entanglements via a Secondary Equilibrium Approach. Polymers.

[69] Valtasaari L. (1971). The configuration of cellulose dissolved in iron-sodium tartrate. Die Makronwlekulare Chem..

[70] Ardizzone S., Fregonara G., Trasatti S. (1990). “Inner” and “outer” active surface of RuO_2_ electrodes. Electrochim. Acta.

[71] Liu P., Borrell P.F., Božič M., Kokol V., Oksman K., Mathew A.P. (2015). Nanocelluloses and their phosphorylated derivatives for selective adsorption of Ag^+^, Cu^2+^ and Fe^3+^ from industrial effluents. J. Hazard. Mater..

[72] Sriplai N., Pinitsoontorn S. (2021). Bacterial cellulose-based magnetic nanocomposites: A review. Carbohydr. Polym..

[73] Cheng Y., Ren X., Duan L., Gao G. (2020). A transparent and adhesive carboxymethyl cellulose/polypyrrole hydrogel electrode for flexible supercapacitors. J. Mater. Chem. C.

[74] Sun Z., Thielemans W. (2023). Interconnected and high cycling stability polypyrrole supercapacitors using cellulose nanocrystals and commonly used inorganic salts as dopants. J. Energy Chem..

[75] Xu H., Cui L., Pan X., An Y., Jin X. (2022). Carboxymethylcellulose-polyaniline/carbon nanotube (CMC-PANI/CNT) film as flexible and highly electrochemical active electrode for supercapacitors. Int. J. Biol. Macromol..

[76] Huang M., Li L., Ai Z., Gao X., Qian J., Xu H., Su X., Wu J., Gao Y. (2022). One-Step Fabrication of Ice-Templated Pure Polypyrrole Nanoparticle Hydrogels for High-Rate Supercapacitors. ACS Appl. Nano Mater..

[77] Buyanov A.L., Gofman I.V., Bozhkova S.A., Saprykina N.N., Kochish A.Y., Netyl’ko G.I., Khripunov A.K., Smyslov R.Y., Afanas’ev A.V., Panarin E.F. (2016). Composite hydrogels based on polyacrylamide and cellulose: Synthesis and functional properties. Russ. J. Appl. Chem..

[78] Dmitriev I., Kuryndin I., Bobrova N., Smirnov M. (2015). Swelling behavior and network characterization of hydrogels from linear polyacrylamide crosslinked with glutaraldehyde. Mater. Today Commun..

[79] Ballauff M., Borisov O. (2006). Polyelectrolyte brushes. Curr. Opin. Colloid Interface Sci..

[80] Borisov O.V., Birshtein T.M., Zhulina E.B. (1991). Collapse of grafted polyelectrolyte layer. J. Phys. II.

[81] Ross R.S., Pincus P. (1992). The polyelectrolyte brush: Poor solvent. Macromolecules.

[82] Zhulina E.B., Borisov O.V. (1996). Polyelectrolytes Grafted to Curved Surfaces. Macromolecules.

[83] Borisov O.V., Zhulina E.B. (2018). Conformations of polyelectrolyte molecular brushes: A mean-field theory. J. Chem. Phys..

[84] Manning G.S. (1969). Limiting Laws and Counterion Condensation in Polyelectrolyte Solutions I. Colligative Properties. J. Chem. Phys..

