# Supplementary material for: A Polyelectrolyte Colloidal Brush Based on Cellulose: Perspectives for Future Applications
Source: Polymers (Basel). 2023 Nov 25;15(23):4526. doi: 10.3390/polym15234526 (PMC10708233; doi:10.3390/polym15234526)
Supplement: Supplementary file 1 [file polymers-15-04526-s001.zip › polymers-2677962-supplementary.pdf]

Polymers

ELECTRONIC SUPPLEMENTARY INFORMATION FOR

A Polyelectrolyte Colloidal Brush Based on Cellulose: Perspectives for Future Applications

Michael A. Smirnov[1,*], Vitaly K. Vorobiov[1], Veronika S. Fedotova[1], Maria P. Sokolova[1], Natalya V. Bobrova[1], Nikolay N. Smirnov[1] and Oleg V. Borisov[1,2,*]

[1] Institute of Macromolecular Compounds, Russian Academy of Sciences, V.O. Bolshoi Pr. 31, 199004 St. Petersburg, Russia; vrbvrbvrb@mail.ru (V.K.V.), fedotova.veronicka2016@yandex.ru (V.S.F.), pmarip@mail.ru (M.P.S.), natalia.bobrova.60@mail.ru (B.N.V.), rambow@inbox.ru (N.N.S);

[2] Institut des Sciences Analytiques et de Physico-Chimie pour l'Environnement et les Matériaux (IPREM), UMR 5254 CNRS/UPPA, 64053 Pau, France.

* Correspondence: smirnov_michael@mail.ru (M.A.S.), oleg.borisov@univ-pau.fr (O.V.B.).

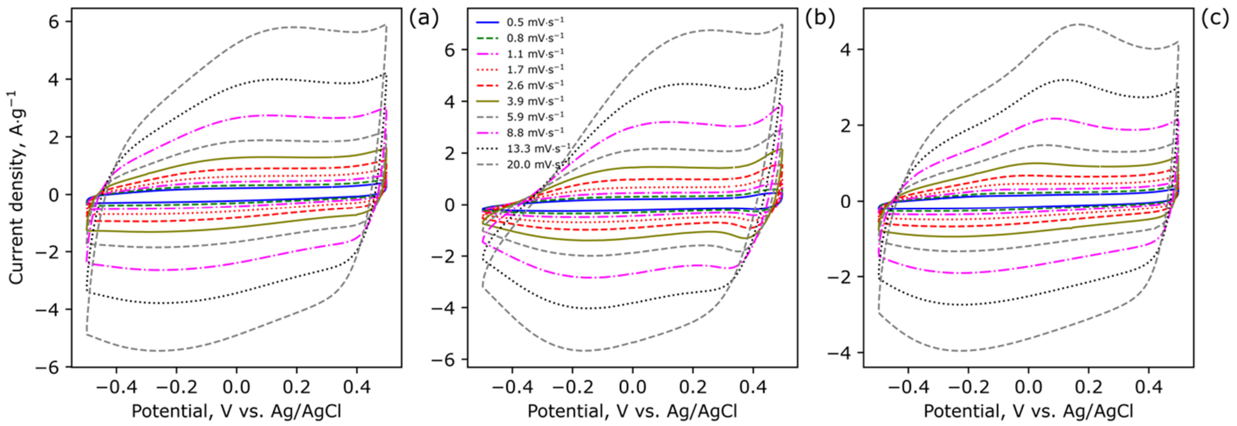

Figure S1. Cyclic voltammetry curves for CNF/PPy (**a**) and CNF-PAA/PPy (**b**) electrodes synthesized using $FeCl_3$ as an initiator and CNF-PAA/PPy electrode synthesized using $(NH_4)_2S_2O_8$ at different scan rates (**c**).
